# Loss of ABHD5 promotes the aggressiveness of prostate cancer cells

**DOI:** 10.1038/s41598-017-13398-w

**Published:** 2017-10-12

**Authors:** Guohua Chen, Guoli Zhou, Siddhesh Aras, Zhenhui He, Stephanie Lucas, Izabela Podgorski, Wael Skar, James G. Granneman, Jian Wang

**Affiliations:** 1 Department of Pathology, Wayne State University, Detroit, MI 48201 USA; 2 Biomedical Research Informatics Core, Clinical and Translational Sciences Institute, Michigan State University, East Lansing, MI 48824 USA; 3 Center for Molecular Medicine and Genetics, Wayne State University, Detroit, MI 48201 USA; 4 Department of Laboratory Medicine, Foshan University Medical College, Foshan, Guangdong 528000 China; 5 Department of Pharmacology, Wayne State University, Detroit, MI 48201 USA; 6 Cardiovascular Research Institute, Wayne State University, Detroit, MI 48201 USA

## Abstract

The accumulation of neutral lipids in intracellular lipid droplets has been associated with the formation and progression of many cancers, including prostate cancer (PCa). Alpha-beta Hydrolase Domain Containing 5 (ABHD5) is a key regulator of intracellular neutral lipids that has been recently identified as a tumor suppressor in colorectal cancer, yet its potential role in PCa has not been investigated. Through mining publicly accessible PCa gene expression datasets, we found that ABHD5 gene expression is markedly decreased in metastatic castration-resistant PCa (mCRPC) samples. We further demonstrated that RNAi-mediated ABHD5 silencing promotes, whereas ectopic ABHD5 overexpression inhibits, the invasion and proliferation of PCa cells. Mechanistically, we found that ABHD5 knockdown induces epithelial to mesenchymal transition, increasing aerobic glycolysis by upregulating the glycolytic enzymes hexokinase 2 and phosphofrucokinase, while decreasing mitochondrial respiration by downregulating respiratory chain complexes I and III. Interestingly, knockdown of ATGL, the best-known molecular target of ABHD5, impeded the proliferation and invasion, suggesting an ATGL-independent role of ABHD5 in modulating PCa aggressiveness. Collectively, these results provide evidence that ABHD5 acts as a metabolic tumor suppressor in PCa that prevents EMT and the Warburg effect, and indicates that ABHD5 is a potential therapeutic target against mCRPC, the deadly aggressive PCa.

## Introduction

Prostate cancer (PCa) is the most common solid organ malignancy in men and the second leading cause of cancer-related death reported in industrialized countries^1^. Because PCa belongs to a class of hormone-dependent malignancies, androgen deprivation therapy (ADT) has been employed as the frontline approach for its therapeutic intervention for over 70 years^2^. However, despite initial responsiveness to ADT, the majority of the treated patients will relapse within two years, and further develop metastatic and castration-resistant PCa (mCRPC), the most aggressive and deadly form of PCa^3,4^. Presently, how PCa cells acquire the ability to grow and invade in the absence of hormonal stimulation is not well understood.

Cancer initiation and progression involve derangement of cellular metabolism^5^. For example, many cancer cells prefer partial catabolism of glucose in cytosol to complete oxidation in mitochondria, even in the presence of abundant oxygen. The phenomenon of aerobic glycolysis, termed the Warburg effect^6^, is a central feature of cancer cell metabolism that efficiently reserves anabolic building blocks for proliferation and reduces oxidative stress resulting from mitochondrial respiration, thus providing proliferative and survival advantages^7,8^. Notably, PCa cells do not exhibit a switch to aerobic glycolysis until the disease progresses to the mCRPC stage^9–14^, suggesting that there might be important alterations in oxidative metabolism that facilitate PCa progression.

Many cancer cells, including PCa cells, often display marked expansion of lipid droplets (LDs), the intracellular organelles that serve as the storage depots for neutral lipids such as triacylglycerols (TAGs)^15,16^. LDs control the availability of free fatty acids (FA), which act both as signaling molecules and fuels for mitochondrial oxidative metabolism when glucose is in short supply^17,18^. Biochemically, mobilization of FAs from LDs is achieved through the process of lipolysis in which FAs are made available from hydrolysis of TAGs by triacylglycerol lipases. Alpha-beta Hydrolase Domain Containing 5 (ABHD5) is an activating coenzyme for Adipose Triglyceride Lipase (ATGL), the rate-limiting lipase in numerous tissues^18^. In humans, loss-of-function mutations of the ABHD5 gene lead to Chanarin-Dorfman Syndrome, a rare hereditary disorder that is characterized by excessive deposition of neutral lipids in multiple organs^19,20^. In mice, ablation of ABHD5 gene in liver decreases respiration and increases glycolysis, conferring a metabolic state reminiscent of the Warburg effect^21^. These data suggest that alteration of ABHD5 might also influence cancer metabolism. Recently, pioneering work by Ou *et al*. identified ABHD5 as a novel tumor suppressor in colorectal cancer^22^. Using a combination of genetically engineered mouse models and molecular cellular approaches, these authors discovered that intestine-specific ablation of ABHD5 dramatically promotes metastatic progression of colorectal tumor, by downregulation of AMPK/p53 signaling and thereby promoting the epithelial to mesenchymal transition (EMT)^22^. Interestingly, it was later found that elevation of ABHD5 in tumor-associated macrophages promotes colorectal tumor growth^23^, indicating a cell-type-specific functional role of ABHD5. Thus, although ABHD5 established as a tumor suppressor in colon cancer, its regulatory roles in other cancer types have not been investigated.

In this study, we analyzed ABHD5 gene expression in the publicly accessible human PCa datasets. Our results showed that ABHD5 is markedly decreased in mCRPC. Using LNCaP cell as a model system, we found that ABHD5 knockdown increased, whereas its overexpression reduced, the aggressiveness of PCa cells. As expected, ABHD5 silencing promoted aerobic glycolysis. Intriguingly, we found ABHD5 silencing reduced expression of specific respiratory chain complexes (RCCs). Thus, our results indicate that ABHD5 suppresses PCa aggressiveness by supporting oxidative metabolism, and suggest that activation of ABHD5 might be useful in treatment of advanced PCa.

## Results

### ABHD5 is downregulated in mCRPC

**Figure 1:**
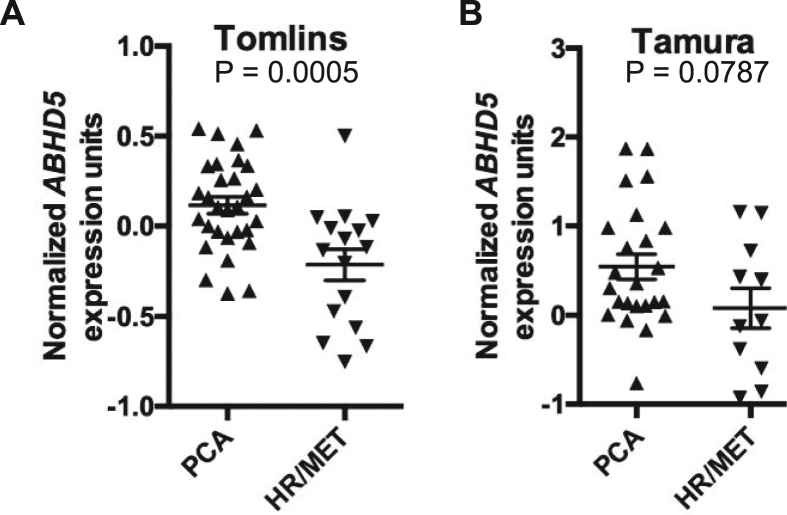
Markedly decreased ABHD5 gene expression in metastatic castration-resistant PCa. Scatter plots demonstrate ABHD5 gene expression in primary (PCA) and hormone-refractory metastatic (HR/MET) prostate cancers in Tomlins (**A**) and Tamura (**B**) datasets.

We first examined the human PCa expression datasets that were published in the Gene Expression Omnibus (GEO) database and found 2 datasets, Tomolins^24^ and Tamura^25^, that contain sufficient mCRPC samples for statistical analyses. In the Tomlins dataset, ABHD5 gene expression was significantly decreased in the mCRPC cases compared to the non-metastatic PCa (p = 0.0005, Fig. 1A). Decreased expression of ABHD5 was also suggested in Tamura dataset, although the statistical significance was marginal due to a larger variance of the data (p = 0.08, Fig. 1B). These results suggest that ABHD5 gene expression is suppressed in mCRPC.

### ABHD5 expression is decreased whereas TAG content is increased in aggressive PCa cells

**Figure 2:**
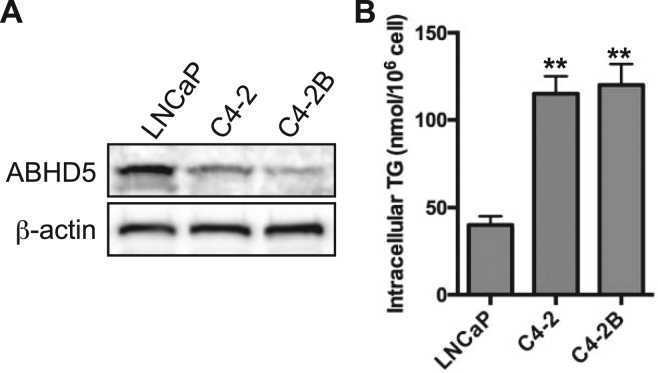
Determination of ABHD5 expression and triacylglycerol content in LNCaP, C4-2 and C4-2B cells. (**A**) Expression level of endogenous ABHD5 was determined in LNCaP, C4-2 and C4-2B cells by Western blotting. β-actin was used as a loading control. (**B**) Intracellular triacylglycerol level in LNCaP, C4-2 and C4-2B cells. Data are presented as mean ± SEM (n = 3) of experiments performed in triplicate. Two tailed t-test, *P < *0.01*.

LNCaP, C4-2 and C4-2B are isogenic PCa cell lines differing in the degrees of tumorigenicity and androgen dependence^26,27^. As compared to low metastatic and androgen-sensitive LNCaP cells, C4-2 and C4-2B cells are highly metastatic and androgen-insensitive^26,27^. Compared to low-metastatic LNCaP cells, ABHD5 protein levels were consistently lower (Fig. 2A) and intracellular TAG higher (Fig. 2B) in C4-2 and C4-2B cells. The reciprocal relationship between ABHD5 expression and TAG accumulation among these PCa cells lines suggested a possible role of ABHD5 in reducing the aggressive phenotype of PCa cells.

### Knockdown of ABHD5 promotes the epithelial to mesenchymal transition of PCa cells

**Figure 3:**
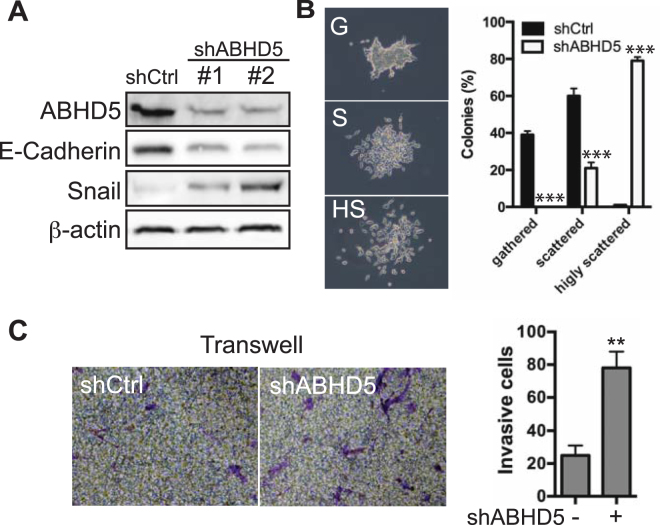
Effects of ABHD5 silencing on the migration and invasion of LNCaP cells. (**A**) Expression level of ABHD5, E-Cadherin, Snail and β-actin proteins in shControl and shABHD5 cells. (**B**) Enhanced cell scattering of shABHD5 cells. shControl and shABHD5 cells were seeded at low density and grown for 2 weeks. Cell colonies (>100 cells/colony) were analyzed under the microscope and sorted into the following groups according to morphology: *gathered (G)*, cells attached to each other tightly; *scattered (S)*, cells scattered but attached loosely; *highly scattered (HS)*, cells detached from each other. Percentages of colonies from each group are plotted in the left panel. Data are presented as mean ± SEM (n = 3) of experiments performed in triplicate. Two-tailed t-test, ***P < *0.001*. Representative images of cell colonies are shown on the right. (**C**) Enhanced cell invasion of shABHD5 cells. A representative image of Transwell invasion assay is shown for each group, respectively, (left panels). Numbers of invasive cell are plotted on the right. Data are presented as mean ± SEM (n = 3) of experiments performed in triplicate. Two-tailed t-test, **P < *0.01*.

Next, we investigated whether altering ABHD5 expression influences PCa cell behavior. We first used shRNA to silence ABHD5 expression in LNCaP cells, which express relatively high levels of ABHD5 (Fig. 2A). LNCaP cells were infected with lentiviral vectors expressing either ABHD5-targeting shRNA (shABHD5) or scramble control (shCtrl), and pools of cells were tested following selection with puromycin. Western blotting demonstrated that shABHD5 with two different shRNA sequences both reduced ABHD5 expression by >70% (Fig. 3A). Knockdown of ABHD5 reduced the expression of E-Cadherin, an epithelial marker, and elevated that of Snail, a mesenchymal maker (Fig. 3A), indicating that loss of ABHD5 activates EMT of LNCaP cells. Knockdown of ABHD5 dramatically promoted cell scattering (Fig. 3B): whereas all colonies of shABHD5 cells were scattered and ~80% were scored as highly scattered, ~40% of control colonies were scored as gathered. These results indicated enhanced migration as a result of ABHD5 silencing. Furthermore, Matrigel Boyden chamber analysis showed that knockdown of ABHD5 enhanced cell invasion by ~4 fold (Fig. 3C). Thus, ABHD5 silencing confers an invasive phenotype to LNCaP cells by activating EMT.

### Mechanistic aspects for EMT activation by ABHD5 deficiency

ABHD5 deficiency in colorectal cells downregulates the AMPK/p53 signaling axis, leading to EMT^22^. To test whether this occurs in PCa cells, we first measured the levels of phosphorylated AMPK and total p53 in the LNCaP cells with or without ABHD5 knockdown. The results showed that shABHD5 decreased the expression of phospho-AMPKα and p53 (Supplementary Figure 1), suggesting that ABHD5 silencing downregulates AMPK/p53 signaling in LNCaP cells. PC3 is a prostate cancer cell line deficient of p53^28^. We knocked down ABHD5 in PC3 cells with shRNAs and observed that knockdown of ABHD5 had negligible effects on the expression of E-Cadherin and Snail (Supplementary Figure 2A) and on cell invasion (Supplementary Figure 2B), which suggests that p53 is required for activation of EMT in PCa cells by ABHD5 loss. Thus, EMT activation by ABHD5 deficiency in PCa cells may involve downregulation of AMPK/p53 signaling axis.

### Knockdown of ABHD5 promotes TAG accumulation and aerobic glycolysis

**Figure 4:**
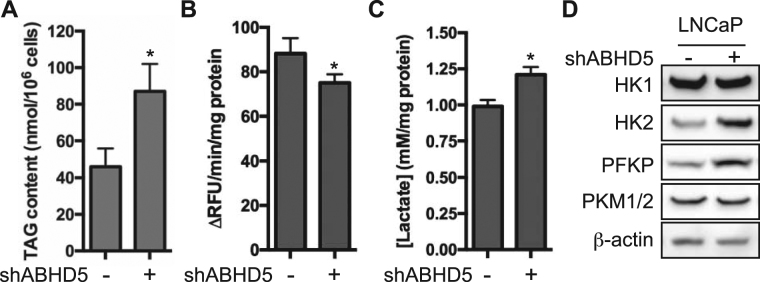
ABHD5 silencing elevates the production of lactic acid and upregulates the expression of key glycolytic enzymes. Intracellular TAG content (**A**), lipase activity (**B**) and lactic acid secretion (**C**) of LNCaP cells. Data are presented as mean ± SEM (n = 3) of experiments performed in triplicate. Two-tailed t-test, *P < *0.05*. (**D**) Protein level of key glycolytic enzymes in LNCaP cells.

A possible mechanism by which loss of ABHD5 function promotes the aggressiveness of PCa cells is by altering cellular metabolism. To test this possibility, we examined whether ABHD5 silencing influences the TAG content of LNCaP cells. The results showed that ABHD5 silencing increased TAG level by ~2-fold (Fig. 4A), consistent with the reduced cellular triglyceride hydrolysis activity (Fig. 4B).

shABHD5 cells were observed to acidify culture media much faster than shControl cells, as indicated by rapid yellowing of culture media. Analysis of culture media showed that shABHD5 cells produced lactic acid at a significantly higher rate than shControl cells (Fig. 4C), indicating enhanced aerobic glycolysis. Consistent with elevation of glycolytic flux, we found that ABHD5 silencing upregulated hexokinase (HK) 2 and phosphofrucotokinase P (PFKP) protein expression without affecting levels of HK1 or pyruvate kinase M1/M2 (PKM1/2) (Fig. 4D). Thus, ABHD5 silencing promotes aerobic glycolysis (the Warburg effect) in LNCaP cells by upregulating key glycolytic enzymes.

### Knockdown of ABHD5 impairs mitochondrial respiration

**Figure 5:**
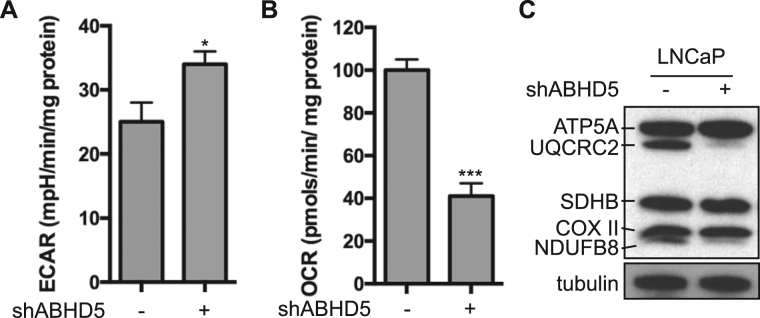
ABHD5 silencing reduces oxygen consumption and downregulates the expression of RCC I and III. Basal extracellular acidification rate (EACR) (**A**) and oxygen consumption rate (OCR) (**B**) of LNCaP cells measured with a Seahorse metabolic flux analyzer. Data are presented as mean ± SEM (n = 3) of experiments performed in triplicate. Two-tailed t-test, *P < *0.05*, ***P < *0.001*. (**C**) Protein levels of respiratory chain complexes of LNCaP cells visualized with Total OXPHOS antibody cocktail in Western Blotting.

To further characterize the metabolic effects of ABHD5 knockdown, we performed metabolic flux measurements on a  XF^e^24 Seahorse analyzer. As expected from the measurement of lactate production, ABHD5 silencing significantly elevated the basal extracellular acidification rate (EACR) (Fig. 5A). In contrast, results further showed that ABHD5 silencing significantly decreased the basal oxygen consumption rate (OCR) (Fig. 5B), indicating reduced mitochondrial respiration. To explore the molecular basis for regulation of mitochondrial respiration by ABHD5, we determined the influence of ABHD5 silencing on the expression of respiratory chain complex (RCC) proteins. Simultaneously probing expression markers of all RCCs using a cocktail of OXPHOS antibodies demonstrated that ABHD5 silencing selectively downregulated RCCs I and III (Fig. 5C).

### Overexpression of ABHD5 inhibits the aggressiveness of PCa cells

**Figure 6:**
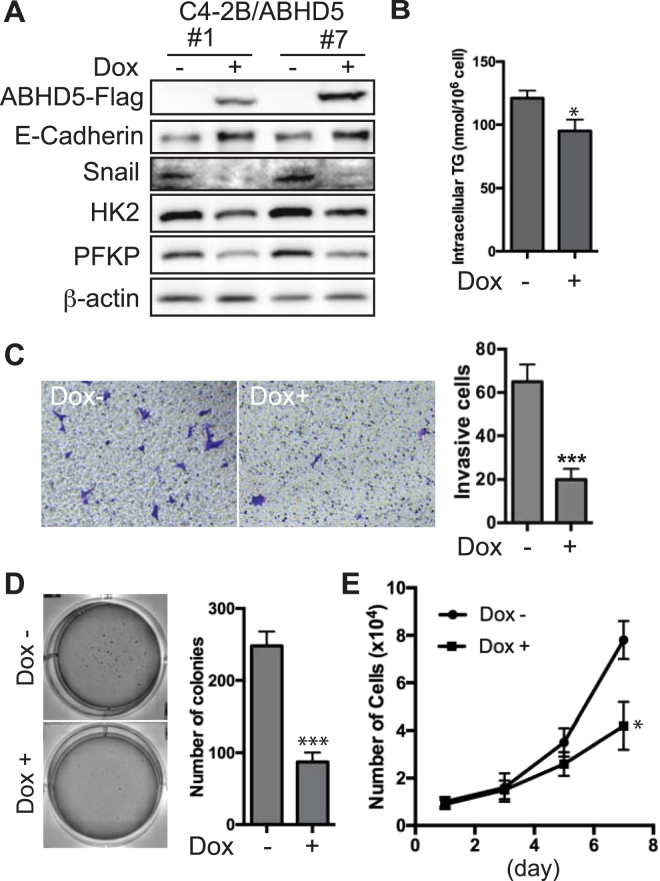
Effects of ectopic ABHD5 overexpression on the proliferation and invasion of C4-2B cells. (**A**) Expression level of ABHD5-Flag, E-Cadherin, Snail, HK2, PFKP and β-actin proteins in C4-2B/ABHD5 cells treated with or without doxycycline (dox). (**B**) Level of intracellular TAG content in C4-2B/ABHD5 cells treated with or without dox. Two-tailed t-test, *P < *0.05*. (**C**) Repression of cell invasion by ABHD5. A representative image of Transwell invasion assay is shown for each group, respectively, on the left. Numbers of invasive cell are plotted on the right. Data are presented as mean ± SEM (n = 3) of experiments performed in triplicate. Two-tailed t-test, ***P < *0.001*. (**D**) Repression of colony formation by ABHD5. A representative image of soft agar assay is shown for each group, respectively, on the left. Number of colonies is plotted on the right. Data are presented as mean ± SEM (n = 3) of experiments performed in triplicate. Two-tailed t-test, ***P < *0.001*. (**E**) Repression of cell proliferation by ABHD5. Growth curve is plotted for C4-2B cells grown in the presence or absence of doxycycline. Data are presented as mean ± SEM (n = 3) of experiments performed in triplicate. Two-way ANOVA test, *P < *0.05*.

To test whether increased ABHD5 expression could antagonize the aggressiveness of PCa cells, we overexpressed ABHD5 in C4-2B, the cell line expressing relatively low level of ABHD5 (Fig. 2A), using a doxycycline (dox)-responsive vector^29^. Following viral vector infection and drug-resistance selection, we identified ABHD5-overexpressing cells and designated these cells as C4-2B-ABHD5 cells. As shown in Fig. 6A, a 2-day dox treatment markedly induced Flag-tagged ABHD5 expression, concomitantly decreasing the expression of Snail, HK2 and PFKP, but increasing that of E-Cadherin in two individual clones. Further functional characterization of C4-2B-ABHD5 cells showed that overexpression of ABHD5 significantly reduced intracellular TAG contents (Fig. 6B), cell invasion (Fig. 6C), colony formation (Fig. 6D) and cell proliferation (Fig. 6E). Thus, overexpression of ABHD5 was sufficient to inhibit the aggressiveness of PCa cells.

### Knockdown of ATGL impedes proliferation and invasion of PCa cells

**Figure 7:**
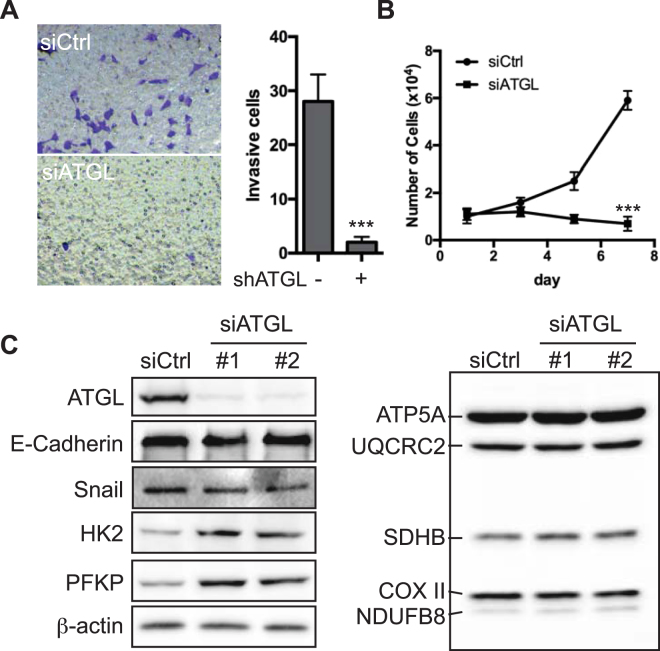
Knockdown of ATGL impedes proliferation and invasion of LNCaP cells. (**A**) ATGL knockdown strongly decreases cell invasion. A representative image of Transwell invasion assay is shown for each group on the left. Numbers of invasive cells are plotted on the right. Two-tailed test, ***P < 0.001. (**B**) ATGL knockdown strongly slows cell proliferation. Growth curve is plotted for LNCaP cells treated with or without the siRNA against ATGL. Two-way ANOVA test, ***P < 0.001. (**C**) Protein expression of ATGL, E-Cadherin, Snail, HK2, PFKP, RCCs and β-actin, analyzed by Western blotting.

ABHD5 is well characterized as a coactivator of ATGL, the rate-limiting enzyme of lipolysis in numerous cell types. To test whether ATGL mediates the effects of ABHD5 on PCa cell aggressiveness, we studied the effects on cell proliferation and invasion by ATGL knockdown with siRNA oligos in LNCaP cells. Surprisingly, we found that knockdown of ATGL severely slowed cell proliferation (Fig. 7A) and inhibited cell invasion (Fig. 7B). Knockdown of ATGL also had negligible effects on the expression of E-Cadherin and Snail (Fig. 7C). Nonetheless, knockdown of ATGL upregulated the expression of HK2 and PFKP (Fig. 7C, left), without affecting the expression of RCCs (Fig. 7C, right). Thus, these results suggest that ABHD5 and ATGL may diverge in the molecular pathways for modulating PCa cell aggressiveness and certain aspects of the metabolic phenotype.

## Discussion

Despite the initial effectiveness of standard therapy, the majority of PCa patients relapse and develop the incurable and deadly mCRPC. Reprogramming of cellular metabolism has been linked to the progression of prostate tumors^30,31^, raising the possibility that new therapeutic targets might be discovered in the pathways that support aberrant metabolism in PCa. In this study, we examined the role of ABHD5, a lipase co-activator and newly identified tumor suppressor^22^, in PCa progression. Our results indicate that ABHD5 gene expression is reduced in mCRPC, and that ABHD5 is critical in maintaining oxidative cellular metabolism that suppresses the aggressiveness of PCa cells. These results provide strong evidence to support ABHD5 as a novel target against PCa progression.

The marked accumulation of intracellular lipid droplets is a well-known morphological feature of PCa^16^, and such accumulation is often more pronounced in metastatic PCa cells^32,33^. While lipogenesis, the biosynthetic arm of lipid metabolism, is upregulated in PCa and likely promotes PCa oncogenesis^34^, how the TAG catabolism of stored lipids influences PCa progression has not been investigated. The present work shows that loss of ABHD5 function promotes EMT (Fig. 3) and TAG accumulation (Fig. 4) of LNCaP cells, suggesting an important role of ABHD5 in reducing the aggressiveness of PCa cells. Consistent with this conclusion we found that ectopic overexpression of ABHD5 attenuates both proliferation and invasion of C4-2B cells (Fig. 6). These findings support a novel anti-oncogenic role of ABHD5 in PCa and suggest that activation of ABHD5 function might have utility against advanced PCa.

The molecular mechanism by which ABHD5 deficiency activates EMT was explored in the present study. A comprehensive study by Ou *et al*. revealed that loss of ABHD5 in colorectal cancer cells represses AMPK/p53 signaling, leading to activation of EMT^22^. Consistent with these observations, we found that silencing ABHD5 decreased the expression of pAMPKα and p53 in LNCaP cells (Supplementary Figure 1). In addition, we observed that p53-null PC3 cells were refractory to the EMT activation by ABHD5 knockdown (Supplementary Figure 2), suggesting a critical role of p53 for mediating the modulation of EMT status by ABHD5 in PCa cells.

Advanced PCa cells adopt the Warburg phenotype to support metastatic progression and androgen resistance^35–37^. Our results demonstrate that ABHD5 silencing strongly promotes aerobic glycolysis as revealed by increased production of lactic acid (Fig. 4C) and by upregulated expression of the rate-limiting glycolytic enzymes HK2 and PFKP (Fig. 4D) in shABHD5 cells. It was reported that HK2 is a key mediator for promoting mCRPC progression under conditions of PTEN and p53 loss^36^. Thus, the ABHD5/HK2 axis identified here suggests that ABHD5 prevents aerobic glycolysis and thereby reinforces tumor suppression by PTEN and p53. Our results also demonstrate that ABHD5 silencing greatly decreases mitochondrial respiration in PCa cells (Fig. 5B), similar to the effects observed in colon cancer cells^22^ and other normal cells^21^. Interestingly, ABHD5 silencing dramatically downregulated the expression of RCC I and III enzymes, which indicates an unanticipated role of ABHD5 in modulating the electron transport chain (ETC). It is not currently known how ABHD5 modulates ETC gene expression; however, it is possible that fatty acids generated from ABHD5-mediated lipolysis act either as metabolic fuels to boost mitochondrial oxidative metabolism or as signaling molecules to promote mitochondrial biogenesis^38^. Taken together, our results indicate that ABHD5 is required for both inhibiting aerobic glycolysis and maintaining mitochondrial respiration, which together prevents the Warburg effect.

The TAG lipase ATGL is the best-known molecular target of ABHD5. However, our results revealed that knockdown of ATGL failed to mimic the effects of ABHD5 with respect to cell proliferation and invasion (Fig. 7A and B), and expression of EMT markers (Fig. 7C). In this regard, although loss of ABHD5 and ATGL function increases TAG accumulation in numerous tissues, defects in these genes give rise to distinct phenotypes^19,39,40^, indicating that ABHD5 regulates lipases and pathways that are independent of ATGL. Our results, which are consistent with the early observations made in colorectal cancer cells^22^, provide further evidence that ABHD5 modulates certain aspects of cancer aggressiveness independently of ATGL.

In summary, our findings strongly support ABHD5 as a suppressor of the Warburg effect and the aggressive progression of PCa cells. Given that ABHD5 activity can be directly modulated by endogenous and synthetic allosteric ligands^41^, our findings suggest that ABHD5 might be developed as a new therapeutic target for advanced PCa.

## Methods

### Cell culture, plasmid construction, viral packaging and cell line establishment

Human prostate cancer cell lines LNCaP (ATCC), C4-2 and C4-2B (Urocor Inc. Oklahoma) were maintained in RPMI 1640 medium supplemented with 10% fetal bovine serum (FBS) and 100 U of penicillin/ml, and 0.1 ng of streptomycin/ml. HEK293FT (Invitrogen) cells were maintained in DMEM medium supplemented with 10% fetal bovine serum (FBS) and 100 U of penicillin/ml, and 0.1 ng of streptomycin/ml.

For construction of lentiviral vector for ectopic overexpression of ABHD5, the DNA fragment encoding full-length human ABHD5 protein was PCR-amplified with the primer pair, 5′-GGGGACAAGTTTGTACAAAAAAGCAGGCTATCGATGCCACCATGGCGGCGGAGGAGGAG-3′ and 5′-TTTGTCGACTCACTTGTCATCGTCGTCCTTGTAGTCTCCGCCCCCGTCCACAGTGTCGCA-3′, and cloned into pInducer20^29^ with gateway cloning technology (Invitrogen). The resultant expression vector was designated as pInducer20-ABHD5. For knockdown of ABHD5, the pLKO.puro lentiviral small-hairpin RNA vectors against human ABHD5 (TRCN0000052020 and TRCN0000299891) was obtained from Sigma. For knockdown of ATGL, the siRNA oligos (SASI_Hs01_00225605 and SASI_Hs01_00225606) were obtained from Sigma and transfected into cells with RNAiMAX transfection reagent (Invitrogen).

For lentiviral packaging, pInducer20 or pLKO.puro vectors was cotransfected with pMD2.G and psPAX2 packaging vectors into HEK293FT cells using lipofectamine 2000 (Invitrogen). 48 h post transfection, virus-containing culture media were harvested and cell debris were removed by passing through 0.45 um filters.

For stable overexpression of ABHD5, C4-2B cells were infected with pInducer20-ABHD5 lentivirus, and selected with 500 ug G418/ml. For stable knockdown of ABHD5, LNCaP cells were infected with pLKO.puro-ABHD5, and selected with 2 ug puromycin/ml. The drug-resistant cells were validated for altered ABHD5 expression by Western blotting.

### Measurements of metabolic fluxes and cellular metabolites

To measure intracellular triacylglycerol, cells were harvested and suspended in 5% NP-40 aqueous solution. Cell extracts were prepared with 2 cycles of heating (95** °**C, 5 min) and cooling (on ice, 5 min) followed by a centrifugation at 12,000 g for 5 min. Cellular triacylglycerol contents were determined with Triglyceride reagent (#T2449, Sigma) on a microplate reader (BMG Labtech) according to the manufacturer’s specifications.

The method of lactic acid measurement was adapted from Brandt *et al*.^42^. In brief, appropriate amount of culture media was incubated at RT for 30 min in a final 100 ul of reaction mix containing 160 mM Tris-Hydrozine (pH 9.0), 2.5 mM NAD^+^, 0.01% BSA and 8 U lactate dehydrogenase. The amount of lactic acid was extrapolated from a standard curve based on the Ab340 reading recorded on a microplate reader (BMG Labtech).

To measure EACR and OCR, 40,000 cells were plated on polylysine coated XF^e^24 extracellular flux plates a day prior to the assay. On the day of analysis, the adherent cells were washed and fresh media were added. Basal oxygen consumption rate (OCR) was measured along with the Extracellular acidification (ECAR) on a Seahorse XF Extracellular Flux Analyzer (Seahorse Bioscience).

### Determination of cell growth and invasion

Cell growth curves were determined as previously described^43^.

To measure cellular capacity for anchor-independent growth, the soft-agar colony formation assay was performed. In brief, 5,000 cells were seeded in the top layer of the agar media containing 0.35% Nobel agar, 1x RPMI 1640 and 10% FBS, and laid on the bottom layer of the agar media containing 0.75% Nobel agar in a 6-well plate. Cells were fed with complete growth media every 3 days. Following cell growth for 3 weeks, cell colonies were stained with crystal violet (0.005% in PBS) and quantified under a microscope.

To measure cellular capacity for invasion, the Boyden chamber Matrigel invasion assay was performed. In brief, cells were seeded in the serum-free media in 8 um Boyden Chamber (Corning) coated with Matrigel (BD Bioscience), and the chamber was placed inside a bottom plate containing complete growth media as migratory attractants. Following 24 h incubation, invasive cells retained on the filter were fixed with 1% paraformaldehyde and stained with 1% crystal violet, and quantified under a microscope.

### Western blotting

Cells were washed twice in phosphate-buffered saline (PBS) and lysed in cytoplasmic lysis buffer (25 mM Tris-HCl pH 7.5, 40 mM NaCl, 1% Triton X-100). Protein concentrations were determined with the Bradford reagent (Bio-Rad). Cell lysates (30 ug) were resolved by sodium dodecyl sulfate-polyacrylamide gel electrophoresis (SDS-PAGE), and proteins were transferred onto nitrocellulose filters. The blots were saturated with 5% nonfat milk and probed with antibodies against ABHD5^38^, E-Cadherin (#610181, BD Biosciences, 1:2000), Snail (#3879, Cell Signaling, 1:500), p53 (#AHO0152, Invitrogen, 1:1000), phospho-AMPKα (#2535, Cell Signaling, 1:1000), HK1 (#2024, Cell Signaling, 1:1000), HK2 (#2867, Cell Signaling, 1:1000), PFKP (#8164, Cell Signaling, 1:1000), PKM1/2 (#3190, Cell Signaling, 1:1000), ATGL (#55190-1-AP, Proteintech, 1:1000), FLAG (#F3165, Sigma, 1:1000), β-actin (A2066, Sigma, 1:1000), Total OXPHOS Human Antibody Cocktail (ab110411, Abcam, 1:1000) or α-tubulin (ab52866, Abcam, 1:1000). Following a wash with PBST (PBS containing 0.1% Tween 20), the blots were incubated with horseradish peroxidase-coupled goat anti-rabbit or –mouse immunoglobulin G (Sigma, 1:5000). The immunolabeled protein bands were detected by enhanced chemiluminescence (ECL) method (Perkin Elmer).

### Cellular lipase activity assay

The method for measurement of cellular lipase activity was adapted from Ou *et al*.^22^. The cellular lipase activity was determined by measuring the rate of fluorescence production (λ_ex_/λ_em_ = 355/460 nM) from fluorogenic ester substrate 4-methylumbelliferyl heptanoate (MUH) by cell homogenates. Briefly, Cells were washed with phosphate buffered saline and then homogenized in the buffer (50 mM Tris-HCl, pH 7.4, 250 mM Sucrose, 1 mM EDTA) by sonication. The assay reaction was initiated by forming the 100 μl reaction mix containing 20 mM Tris-HCl pH 8.0, 1.5 μM MUH, 100 μg cell homogenates and 1 mM EDTA in a 96-well microplate. The changes of fluorescence were monitored in the kinetic mode for 5 minutes on a CLARIOstar microplate reader (BMG Labtech). The lipase activities were expressed as ΔRFU/min/mg protein (RFU = relative fluorescence units).

### Bioinformatics analysis for ABHD5 gene expression in human mCRPC datasets

Two publically available gene expression profiling datasets of prostate cancer, the Tomlins (accession number: GSE6099) and the Tamura (accession number: GSE6811), were retrieved from the NCBI Gene Expression Omnibus (GEO) database (https://www.ncbi.nlm.nih.gov/gds). The Tomlins and the Tamura datasets contain 30 and 24 primary prostate cancer tissues as well as 16 and 11 hormone refractory and metastatic cancer tissues, respectively. For multiple probes corresponding to ABHD5 gene, only the probe closest to the 3′ end of ABHD5 gene full-length mRNA was used based on the available corresponding EST sequences where the probes were derived. Finally, the probe ID labeled as “Hs6-13-4-8” in the Tomlins dataset and as “7562” in the Tamura dataset, the corresponding ABHD5 gene expression values, and the sample source information were abstracted for further analysis. The gene expression values in both datasets were presented as log2-transformed signal intensity ratio (Cy5/Cy3). For each microarray dataset, we classified the samples with both hormone refractory and metastasis as Cases and the ones sampled from primary prostate cancer tissues without metastasis as Controls. After checking the normality of ABHD5 gene expression distribution and the equality of variances between case and control groups in each dataset, a t-test was applied to compare mean difference of ABHD5 expression between two groups. The statistical significance level, α, was set as 0.05 for a two-sided test. The final results were summarized as mean of the log2(ratio) and its corresponding standard error (SE), and further visualized with a scatterplot.

### Statistical analysis

Statistical analyses were performed with SAS v9.4 (SAS Institute, Cary, North Carolina) and GraphPad Prism 6 software (GraphPad Software, La Jolla, CA). Data are presented as means ± SE. Statistical significance between two groups was determined by unpaired *t*-test. Comparison between cell growth curves was performed using a two-way ANOVA.

### Data availability

All data generated and analyzed during this study are included in this published article.

## Electronic supplementary material

Supplementary Information
