# Supplementary material for: Loss of ABHD5 promotes the aggressiveness of prostate cancer cells
Source: Sci Rep. 2017 Oct 12;7:13021. doi: 10.1038/s41598-017-13398-w (PMC5638841; doi:10.1038/s41598-017-13398-w)

Loss of ABHD5 promotes the aggressiveness of prostate cancer cells

Guohua Chen[1], Guoli Zhou[2], Siddhesh Aras[3], Zhenhui He[4], Stephanie Lucas[1], Izabela Podgorski[5], Wael Skar[1], James G. Granneman[3] and Jian Wang[1,6,*]

Department of Pathology[1], Center for Molecular Medicine and Genetics [3], Department of Pharmacology[5], and Cardiovascular Research Institute[6], Wayne State University, Detroit, MI 48201 USA

[2] Biomedical Research Informatics Core, Clinical and Translational Sciences Institute, Michigan State University, East Lansing, MI 48824 USA

[4] Department of Laboratory Medicine, Foshan University Medical College, Foshan, Guangdong 528000, China

*** To whom correspondence should be addressed:**

Email: jianwang@med.wayne.edu

Phone: 313-577-9433

Fax: 313-972-8025

Supplementary Figure 1. Effects on phospho-AMPKα and p53 protein expression by ABHD5 knockdown in LNCaP cells.

Levels of phospho-AMPKα, p53 and β-actin in the LNCaP cells with or without ABHD5 knockdown were analyzed by Western blotting.

Supplementary Figure 2. Negligible effects on EMT marker expression and cell invasion by ABHD5 knockdown in PC3 cells.

A. Levels of ABHD5, E-Cadherin, Snail and β-actin analyzed by Western Blotting.

B. Cell invasion determined by Boyden Chamber Transwell assay.

Supplementary Figure 1

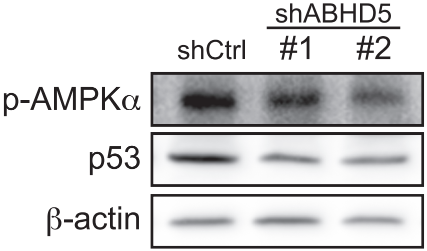

Supplementary Figure 2

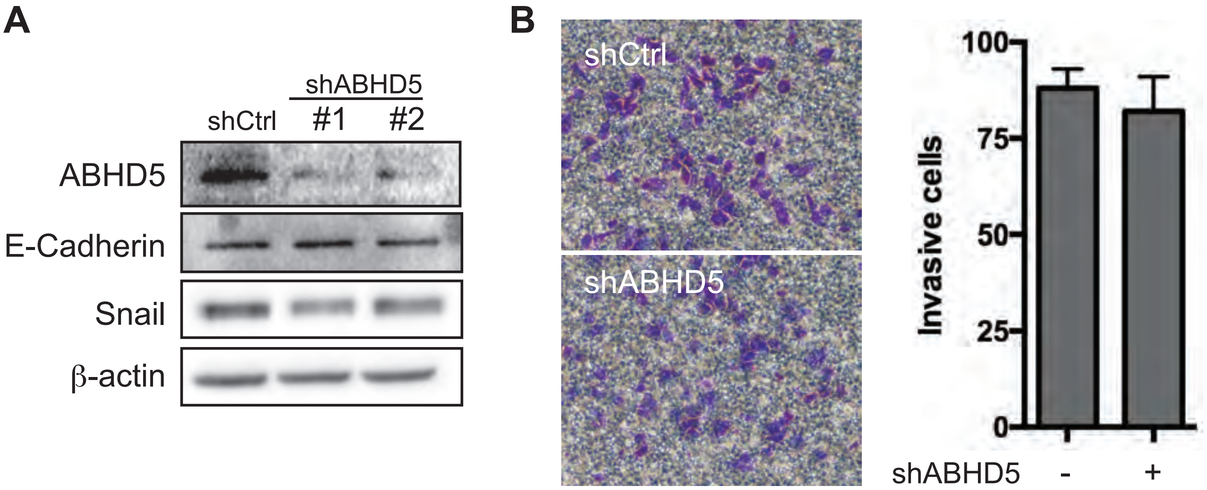

Supplement: Supplementary file 1 — Supplementary Information [file 41598_2017_13398_MOESM1_ESM.pdf]
